# *Clostridioides difficile* as a Zoonotic and Foodborne Pathogen: Reviewing the Global Evidence and South African Data

**DOI:** 10.1002/mbo3.70331

**Published:** 2026-06-23

**Authors:** M. Gouws, P. E. Strydom, D. Rip

**Affiliations:** ^1^ Centre for Food Safety, Department of Food Science, Faculty of AgriSciences Stellenbosch University Stellenbosch South Africa; ^2^ Department of Animal Sciences, Faculty of AgriSciences Stellenbosch University Stellenbosch South Africa

**Keywords:** antibiotic resistance, cattle, food‐producing animal, game, One Health, poultry, ribotype, venison, wildlife

## Abstract

*Clostridioides difficile* infections (CDIs) are becoming increasingly problematic in developing countries, further burdening healthcare systems. In Southern Africa, limited information is available on the prevalence and diversity of *C. difficile* strains in animals, food, and the environment. This review aims to provide insight into global findings on strain diversity, antibiotic resistance, and the epidemiology of *C. difficile* associated with food‐producing animals, wildlife, and food processing environments. The review further explores the zoonotic and foodborne risk potential of the pathogen and includes clinical trends of CDIs within South Africa. It is evident that farm environments and animals play a considerable role in the spread of *C. difficile*. A lack of broad surveillance, incomplete data on CDIs, and the prevalence of *C. difficile* within South Africa hinder an accurate representation of its zoonotic and foodborne potential.

## Graphical abstract

*Clostridioides difficile* infections are increasingly problematic in areas with already burdened healthcare systems. From a One‐Health perspective, a lack of surveillance in developing countries greatly contributes to the limited information available on the zoonotic and foodborne potential of *C. difficile*. Comparing global strain diversity, antibiotic resistance, and epidemiology of *C. difficile* associated with the agricultural‐food sector may provide insight into potential community, animal, and environmental risk areas within South Africa.

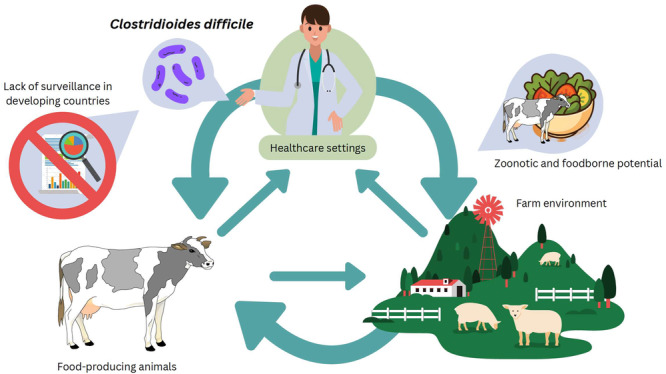

## 1 Introduction

*Clostridioides difficile* is a Gram‐positive, anaerobic, enteric, spore‐forming toxigenic pathogen that can be found in humans, animals, and the environment (Weese 2020; Marcos et al. 2022). Its virulence is linked to its ability to produce toxins, and when this occurs, it can result in infection ranging from symptoms linked to mild diarrhea to life‐threatening pseudomembranous colitis (Kachrimanidou et al. 2019). *C. difficile* infections (CDIs) are difficult to treat due to the bacterium's innate resistance to broad‐spectrum antibiotics.

Over the past decade, a global trend in the increase of community‐acquired *C. difficile* infections (CA‐CDIs) sparked investigation into other possible sources and dissemination routes of the bacterium (Marcos et al. 2022). Normally, patients must have been exposed to the pathogen in a nosocomial setting, taken broad‐spectrum antibiotics or adhered to some of the predisposing factors that increase the likelihood of contracting a CDI. However, an increasing number of patients are now presenting with CDIs, regardless of the typical transmission routes described.

The One‐Health initiative from the World Health Organization emphasizes that the health of humans is interlinked with the health of animals and the environment (Lim et al. 2020). Medical professionals and veterinarians have long been dealing with CDI in separate sectors, but medical professionals may still largely be unaware of the prevalence of toxigenic *C. difficile* present in animals, food, and the environment (Lim et al. 2020).

In South Africa, the epidemiology of *C. difficile* has only been investigated in hospitals, and limited information on potential sources of CA‐CDI is available. Younger patients with no comorbidities are among the categories with CA‐CDI, and these infections are associated with hypervirulent strains (Nana et al. 2020). Globally, alternative sources of *C. difficile* within the community, including those of zoonotic or food origin, are under more frequent investigation (Romano et al. 2018; Weese 2020; Marcos et al. 2021). This review will discuss *C. difficile* as an emerging zoonotic and foodborne pathogen globally. It aims to provide valuable insights into risk factors contributing to community‐associated CDIs in South Africa. Furthermore, it will highlight research gaps in both the global and South African literature and may serve as a guideline for structuring future research aimed at South African conditions.

## 2 *C. difficile* Classification, Epidemiology, and Pathogenesis

*C. difficile* exists in two states: the vegetative form that is sensitive to oxygen and can reproduce in the gastrointestinal tract, and the heat‐stable spore that can be shed through feces, surviving in the environment for long periods of time (Kachrimanidou et al. 2019; Weese 2020). Vegetative cells can appear as rod‐shaped, pleomorphic structures (Taggart et al. 2021). Formally known as *Peptoclostridium* and *Clostridium*, *C. difficile* falls into the class of *Clostridia* spp. (Sandhu and McBride 2018).

There are multiple ways to classify *C. difficile*, with the most common methods used in epidemiology and clinical settings being polymerase chain reaction (PCR)‐ribotyping and toxinotyping, respectively. A ribotype (RT) refers to the classification of a specific strain or subtype of the bacterium based on variations in the sequences of its ribosomal RNA (rRNA) genes. RTs can indicate the presence of certain strains; however, they can be shared between sequence types of the same bacterium (Martínez‐Meléndez et al. 2020). Multilocus sequence typing (MLST) is used to identify sequence types within certain clades, whereas whole‐genome sequencing (WGS) is employed in research where virulence and antibiotic resistance genes, as well as other genomic factors, are considered.

PCR‐ribotyping is the most widely used method to type *C. difficile* in epidemiological studies. This is done by the amplification of the region between the intergenic spacer regions (i.e., 16S and 23S rRNA genes) (Indra et al. 2008; Goyal et al. 2020). Identifying specific RTs associated with various specimen types may help link high‐risk community sources to clinical *C. difficile* infections, enabling better understanding and risk mitigation (Bandelj et al. 2016; Marcos et al. 2021; Abay et al. 2022).

Toxinotyping targets the pathogenicity locus (PaLoc) of *C. difficile*, where the toxin genes are situated (Rupnik and Janezic 2016). The variations are assessed by PCR amplification of the *tcd*A and *tcd*B genes, which encode the two large protein toxins for toxins A and B, respectively. The binary toxin (*cdt*A/*cdt*B) genes are situated outside the PaLoc but is also considered when determining the toxin profiles of *C. difficile*. Furthermore, the virulence of *C. difficile* can be influenced by regulatory genes on the PaLoc (Candel‐Pérez et al. 2019). For instance, the *tcd*C gene is a negative regulator situated on this locus, and increases the production of toxins A and B (Schäffler and Breitrück 2018). Some of these toxin profiles can be associated with hypervirulent strains, such as the RT027, and can provide a direct link to disease risk, and are therefore valuable. The presence of the binary toxin genes is not always essential for disease, but their presence is linked with more hypervirulent strains and is often associated with increased severity of disease and difficult treatment (Kachrimanidou et al. 2019).

### 2.1 Epidemiology

The spread of *C. difficile* is mediated by its dormant spores, while its virulence largely depends on three main *Clostridial* toxins (Zeng et al. 2024). Toxin A, toxin B, and a *C. difficile* binary toxin are considered to be the three main factors of virulence contributing to the severity of CDIs (Voth and Ballard 2005; Li et al. 2020). The production of toxins A and B increases the epithelial permeability of intestinal cells, allowing bacterial translocation and site inflammation, whereas the binary toxin can disrupt the normal cytoskeletal functions of intestinal cells (Di Bella et al. 2016).

The epidemiology of *C. difficile* is further thought to be driven by its innate ability to withstand broad‐spectrum antibiotics (Wickramage et al. 2021). This enables the bacterium to survive in conditions where other microorganisms cannot, thereby outcompeting the natural microbiota. It is well documented that the gut microbiota of patients with CDI differs greatly from that of healthy individuals. They have a severe loss of microbiota diversity, which can be worsened by the use of broad‐spectrum antibiotics (Gonzales‐Luna et al. 2023).

CA‐CDIs are not limited to the classical risk factors associated with nosocomial CDIs. It has become more apparent that the traditional predisposing risk factors, such as prior antibiotic exposure, compromised immune system, or age, do not apply to most CA‐CDI cases over the past 20 years. On the basis of a population study by Khanna et al. (2012), 36% of patients with CDI were younger than 65 years. This is consistent with other studies, where patients were found to be primarily female and younger than 65 years (Thornton et al. 2018), and more recently, the highest rates of CDI were observed in children (Akorful et al. 2025). Noteworthy is that CA‐CDI cases may still be misrepresented, as it is often misdiagnosed in patients (Boven et al. 2023). In South Africa, precise literature on the epidemiology of CA‐CDIs is scarce; however, it has been reported that these infections are more frequent in younger patients (25–45 years of age) (Kullin et al. 2018) with no history of recent antibiotic use (Nana et al. 2020).

*C. difficile* strains can be categorized into clades (1–5) with WGS and MLST. Certain clades may be limited to specific geographical regions, exhibit distinct toxin profiles, and have distinct epidemiological importance. Using this genome‐based classification, RTs such as RT078 (Clade 5) and RT014/020 (Clade 1) have been identified as lacking geographical barriers, where CDIs are considered (Spigaglia et al. 2023). Furthermore, food products from tropical regions are associated with a higher prevalence of *C. difficile* contamination. This high prevalence is not reflected in the low CDI incidence in this region, and therefore theorized that the rich human microbiome of people living in the tropical regions provides protection against CDI even when *C. difficile* spores are ingested (Rodriguez‐Palacios et al. 2020). In contrast, certain RTs are limited to specific geographical regions. For example, RT018 (Clade 1) is considered predominant in Italy, but is not found anywhere else in Europe (Martínez‐Meléndez et al. 2020), whereas RT027 (Clade 2) dominates in North America, and Clade 4 in Asia (Collins et al. 2020).

*C. difficile* RT001 is commonly associated with nosocomial CDIs worldwide, the second most common in South Africa (Kullin et al. 2022), and known for its tendency to cause relapses (Martínez‐Meléndez et al. 2020). It has been isolated from poultry meat and leafy greens, and is the most common toxigenic RT associated with food products in Slovenia (Tkalec et al. 2020). Together with RT010, it was detected in meat, seafood, and vegetable samples in the same study. *C. difficile* RT010 has more frequently been observed in densely populated areas compared with agricultural environments; however, it has also been detected in ready‐to‐eat (RTE) salads and poultry (Tkalec et al. 2020; Bingol et al. 2020; Cautivo‐Reyes et al. 2023).

When considering *C. difficile* association with livestock, the hypervirulent RT027 and RT078 are notable. However, multiple strains continue to contribute to the burden of *C. difficile* within the agricultural sector. *C. difficile* RT014/020 are often listed together, as the ribotyping banding patterns can be difficult to distinguish (Martínez‐Meléndez et al. 2020). It is the most common RT in Europe, the United States of America, and Australia (Foster et al. 2014), and can be found in a broad range of animal hosts. It is also frequently detected in South African CDI patients (Kullin et al. 2022), and one of the few RTs with sufficient evidence to assume zoonotic transmission between swine and human hosts (Knight and Riley 2016).

Even though RT027 is not regularly reported in Africa (Janssen et al. 2016), RT017 accounts for 50% of *C. difficile* detected in South Africa, and is predominantly associated with tuberculosis (TB) patients (Kullin et al. 2017). This RT is resistant to antibiotics frequently used for the treatment of TB; up to 99% of isolates are resistant to rifampicin, and resistance to moxifloxacin has also been detected (Kullin et al. 2017, 2022). Furthermore, despite RT018 being predominantly associated with nosocomial CDI cases in Italy and Japan, it has been found in the environment (Janezic et al. 2012), indicating that this RT is not limited to hospital environments. On the contrary, RT018 has not yet been isolated from food‐producing animals, even though it is thought to have a higher transmission rate than RT078 (Baldan et al. 2015).

Both hypervirulent strains, RT027 and RT078, have only recently been associated with the increase in CA‐CDIs in humans. It is well documented that RT078 is frequently associated with food‐producing animals and wildlife, suggesting zoonotic transmission (Kim et al. 2018; Krutova et al. 2018). Pigs are a reservoir for RT078, as it has been observed to be abundant in swine populations worldwide (Squire et al. 2013; Knetsch et al. 2018; Duarte et al. 2023). Similarly, *C. difficile* RT033 can be considered as an emerging strain of clinical and veterinary importance. It has been detected in food‐producing animals, companion animals, and soil (Kecerova et al. 2019), and has become predominant in swine infections. Even though this RT has also been isolated from symptomatic patients (Androga et al. 2018), predominant RTs in hospital settings have been found to differ widely between countries (Martínez‐Meléndez et al. 2020). Table 1 highlights the RTs detected in animals, food products, and the environment that are of clinical importance.

**Table 1:** *Clostridioides difficile* ribotypes of importance, commonly identified from animal, food, environmental, and clinical origins.

Ribotype	Isolated from	Clinical importance
RT001	Poultry meat,^a^ leafy greens,^a^ clincal^b^	Human–pathogenic^n^
RT010	Seafood,^a^ RTE salad,^a^ chicken carcass^c^	Associated with densely populated areas^j^
RT012	Cattle,^d^ leafy greens,^a^ clinical^b^^,^ e	Most prevalent RT in cattle^d^
RT014	Horse,^c^^,^ f ^,^ g clinical^h^	Human–pathogenic^o^
RT014/020	Poultry meat,^a^ pig,^i^ soil,^j^ clinical^f^	Predominant CA‐CDI associated with clinical settings in Europe and the United States^p^
RT017	Clinical^b^^,^ e	Toxigenic,^q^ hypervirulent,^f^ the highest 30‐day mortality rate than any other RT, mainly associated with CDI in South African tuberculosis patients^r^
RT020	Chicken carcass,^c^ clinical^f^^,^ g	Associated with CA‐CDI^s^
RT027	Processed meats,^k^ cured meats,^k^ poultry,^c^ beef,^l^ sheep,^m^ clinical^b^^,^ e ^,^ g	Hypervirulent^t^
RT033	Pigs, cattle, companion animals,^h^ soil,^h^ clinical	Human–pathogenic^h^
RT078	Beef,^a^ sheep,^m^ goat,^m^ clinical^b^^,^ g	Hypervirulent, associated with zoonotic transmission,^u^ frequently associated with CA‐CDI in younger patients^n^
RT087	Soil,^j^ poultry meat,^a^ chicken carcass^c^	—

Abbreviations: CA‐CDI, community‐acquired *Clostridioides difficile* infection; CDI, *Clostridioides difficile* infection; RT, ribotype.

^a^ Tkalec et al. (2020).

^b^ Adler et al. (2015).

^c^ Bingol et al. (2020).

^d^ Koene et al. (2012).

^e^ Rodriguez et al. (2014a).

^f^ Kiril et al. (2024).

^g^ Freeman et al. (2015).

^h^ Janezic et al. (2018).

^i^ Knight et al. (2015).

^j^ Cautivo‐Reyes et al. (2023).

^k^ Tang et al. (2016).

^l^ Muratoglu et al. (2020).

^m^ Bacheno et al. (2022).

^n^ Martínez‐Meléndez et al. (2020).

^o^ Knight and Riley (2016).

^p^ Foster et al. (2014).

^q^ Imwattana et al. (2019).

^r^ B. Kullin et al. (2017).

^s^ Blau et al. (2023).

^t^ Yuille et al. (2020).

^u^ Knetsch et al. (2018).

Since multiple factors influence high‐risk animal and food groups in the global spread of CDI, it remains necessary to understand *C. difficile* strains present in certain geographical areas within a country. For instance, even with limited information available, it has already been established that the Western Cape of South Africa reports a much higher incidence of CDIs when compared with other provinces (de Jager et al. 2021). However, further investigation into the potential causes of this high incidence is still required, and may lead to the discovery of high‐risk animal, agricultural, environmental, or food groups.

#### 2.1.1 *Infections*: Risk Factors and Treatment

*C. difficile* infections have been one of the leading healthcare‐associated infections worldwide (Balsells et al. 2019). An incidence rate is often calculated to determine the burden of CDIs across countries. The incidence rate of CDI is determined by the number of confirmed cases per 100 000 hospitalizations over a year. South Africa, therefore, has an incidence rate of 53.89, with a recurrence rate of 2.41% (de Jager et al. 2021). This is consistent with the literature, where the incidence rate of CDI ranges from 1.1 to 96.6 cases per year worldwide (Finn et al. 2021; Akorful et al. 2025). The most reported RTs resulting in CDIs include RT001, RT017, RT027, RT014, RT023, RT078, and RT126.

A history of previous antibiotic use is considered the leading risk factor for CDIs. *C. difficile* is an opportunistic pathogen and will proliferate when the natural gut microbiota is disrupted, where hospital‐acquired CDIs are often associated with gut dysbiosis following antibiotic treatment. The natural gut microbiota usually outcompetes *C. difficile* through producing sufficient secondary bile acids (Britton and Young 2014), which protects against its colonization in the gastrointestinal tract of humans and animals.

A point‐prevalence study in Europe, considering only clinical isolates, found that the use of broad‐spectrum penicillins posed a very high risk for CA‐CDIs, whereas fluoroquinolones and cephalosporins were the primary risk for the development of nosocomial CDIs (Viprey et al. 2022). The study did not include data from Germany, as including them could have led to an overrepresentation of Western Europe. However, while this exclusion may have maintained regional balance, it could have limited the provision of new insights into areas previously associated with high rates of CA‐CDI (Weil et al. 2007). In addition, South Africa has a large number of patients receiving antibiotic treatment for TB. Tuberculosis patients are 62% more likely to contract CDI compared with non‐TB patients (de Jager et al. 2021). This puts South Africa in a unique situation compared with European countries and increases strain on an already burdened healthcare system. The gaps in the current knowledge regarding possible community sources of CDI in South Africa are concerning, as the mortality rate for CDI in the elderly can be as high as 90% (Evans and Safdar 2015).

Many studies highlight advanced age as a predisposing factor for CDI (Davies et al. 2020; Nana et al. 2020). Advanced age can coincide with various comorbidities, which may include diabetes, cancer (i.e., chemotherapy), heart disease, dementia, kidney disease, and other chronic diseases (Davies et al. 2020). Other risk factors include a history of surgery (i.e., organ transplant, abdominal surgery), as well as a prior case of CDI associated with a patient. However, in South Africa, a large majority of CA‐CDI cases have been detected in younger patients with no comorbidities, where a history of antibiotic use in the 12 weeks prior to the onset of symptoms is absent (Nana et al. 2020). It has also been observed that these patients are often in contact with infants, farm animals, or reside near farm environments (Ofori et al. 2018). This shift in epidemiology supports the possibility of zoonotic, foodborne, or waterborne transmission of this pathogen.

The treatment regimen for CDI is based on disease severity, patient age, and patient history (e.g., comorbidities, recurrent CDIs). The clinically important antibiotics vancomycin, metronidazole, and fidaxomicin are most commonly used worldwide to treat CDIs (Bass et al. 2013; Stevens et al. 2017; Vitiello et al. 2024). In South Africa, vancomycin and fidaxomicin are preferred for initial, nonsevere cases, and metronidazole is only used in mild cases where neither is available (Nana et al. 2020). Fecal transplants have long been used as a treatment option for severe cases of CDI, which has not only been effective in reducing mortality in at‐risk patients, but also effective for partially treated and initially severe cases of CDI (Cho 2021). For recurrent, severe CDI cases, a fecal transplant is advised, with the best treatment success rate achieved when combined with vancomycin (Baunwall et al. 2020). Even though first‐line agents (i.e., metronidazole and vancomycin) remain effective, as antibiotic exposure increases globally, ongoing monitoring of antibiotic‐resistant (ABR) resistance patterns is necessary to ensure the continued clinical standard treatment remains effective (Thornton et al. 2018; Akorful et al. 2025).

#### 2.1.2 Antibiotic Resistance—Clinical Origin

*C. difficile* is considered resistant to most broad‐spectrum antibiotics (Lucia et al. 2016; Agnoletti et al. 2019). Although *C. difficile* is part of the natural gut microbiota, other bacterial species may naturally outcompete the bacterium in the gastrointestinal tract of healthy individuals (Gonzales‐Luna et al. 2023). The use of broad‐spectrum antibiotics can therefore induce gut dysbiosis through disrupting the bile acid‐modifying activity of the microbiota, and so create an environment in which disease‐causing *C. difficile* can proliferate (Vitiello et al. 2024).

Imwattana et al. (2021) conducted a genotypic evaluation of antimicrobial resistance in *C. difficile*. Worldwide sequence data from clinical, animal, and environmental origins were evaluated using Resfinder, ARGannot, and the CARD 2020 databases to determine genotypic antibiotic resistance. From the 270 sequence types included in the study, 56% were found to be pan‐drug resistant, 8.7% multidrug‐resistant, and 35% resistant to one to two classes of antibiotics. This is consistent with previous literature, where the majority of *C. difficile* show resistance to nearly all commonly prescribed classes of antibiotics (Tang et al. 2016; Webb et al. 2020). Interestingly, compared with *C. difficile* strains from Europe, Asia, and the USA, the prevalence of antibiotic resistance was found to be lower in strains from Australia and New Zealand, and they were therefore used as the reference group in the study (Imwattana et al. 2021).

Even though *C. difficile* often displays multidrug‐resistance, antibiotics are still the main choice of treatment for medical professionals (Vitiello et al. 2024). A history of overprescription of broad‐spectrum antibiotics in developed countries plays a role in disrupting the gut microbiota of the patient and so selects for multidrug‐resistant pathogens (Akorful et al. 2025). For instance, in Slovakia *C. difficile* RT001 and RT176, both associated with more severe clinical presentations, were the most common RTs associated with CDIs. Plankaova et al. (2023) found these RTs to be resistant to fluoroquinolone antibiotics, which are normally used to treat a variety of respiratory and urinary tract infections (National Institute of Diabetes and Digestive and Kidney Diseases 2012).

Most antibiotics carry risk; however, it varies by class and route (Webb et al. 2020). Late generation cephalosporins, fluoroquinolones, carbapenems, and lincosamides are considered high‐risk agents for the development of antibiotic‐associated CDI (Slimings and Riley 2014; Tabak et al. 2019). Furthermore, ampicillin, amoxicillin, cephalosporins, clindamycin, and fluoroquinolones have been specifically associated with an increased risk of CDI during treatment (Nana et al. 2020). The use of vancomycin and metronidazole remains effective (Mihajlov et al. 2024). This has also been documented by Ghosh et al. (2024), where patients frequently treated with clindamycin, fluoroquinolones, and cephalosporins were more susceptible to CA‐CDIs.

Daptomycin and tetracyclines have been thought not to carry any risk of inducing CDIs in humans (Webb et al. 2020). However, a recent study by Zbylicki et al. (2024) indicated that *C. difficile* has the ability to develop resistance to daptomycin. This study induced an in vitro selective environment with a wild‐type *C. difficile* R20291. This strain was subcultured until growth could be observed at a > 16 µg/mL concentration of daptomycin in tryptone‐yeast, whereafter genomic DNA was sequenced to identify mutations responsible for its developed phenotypic resistance. There is currently no other literature suggesting *C. difficile* resistance to daptomycin. Since daptomycin is used to treat methicillin‐resistant *Staphylococcus aureus* and vancomycin‐resistant *Enterococcus* species, it could be effective as a last‐resort *C. difficile* treatment.

Currently, in South Africa, broad‐spectrum antibiotics such as bedaquiline, pretomanid, linezolid, and levofloxacin are prescribed for a 6–9‐month period for patients with rifampicin‐resistant TB (National Department of Health 2023). This may well indirectly contribute to antibiotic resistance in *C. difficile* through prolonged gut microbiome disruption (Bhattarai et al. 2024). For instance, Kullin et al. (2018) observed that 95% of all patient *C. difficile* isolates (RT017) from a South African hospital were considered multidrug‐resistant. This should further urge investigation into the potential of unique epidemiological characteristics of *C. difficile* in South Africa.

#### 2.1.3 Antibiotic Resistance—Nonclinical Origin

Multidrug‐resistant *C. difficile* has greatly influenced the treatment success of clinical and CA‐CDIs (Vitiello et al. 2024). Antibiotic resistance to ampicillin, imipenem, and cefotaxime is common within isolates of meat origin (Muratoglu et al. 2020). The ABR profiles of RT027 and RT078, commonly found within animal meat products in the Middle‐East, show resistance to tetracycline, erythromycin, metronidazole, ciprofloxacin, clindamycin, chloramphenicol, and meropenem (Bacheno et al. 2022). Similar resistance profiles were observed in retail meat and meat products worldwide, where isolates harbored resistance to clindamycin, ciprofloxacin, cefotaxime, and tetracycline (Barezi et al. 2023; Hazarika et al. 2023). Resistance patterns to these antibiotics may be attributed to their historical use in animal agriculture. To understand the complexities of the antibiotic resistance characteristics of *C. difficile*, it is crucial to examine historic and current trends in antibiotic use to mitigate the associated risks.

Clindamycin was routinely prescribed in Europe in the 1970s. However, it was quickly noted that clindamycin contributes to the predisposed risk for the development of CDIs, and the use thereof was reduced worldwide. High levels of resistance to clindamycin and ciprofloxacin remain, and CDI remains frequently associated with clindamycin use (Teng et al. 2019; Miller et al. 2023; Mihajlov et al. 2024). Additionally, resistance to clindamycin, ciprofloxacin, erythromycin, imipenem, moxifloxacin, and tetracycline is typically tied to hypervirulent *C. difficile* RT017 and RT027 (Mihajlov et al. 2024).

According to a scoping review by Singh and Mudey (2021), which focused on ABR mechanisms of *C. difficile*, all European strains were resistant to fidaxomicin, metronidazole, and vancomycin. Imwattana et al. (2021) further observed clear differences in antimicrobial resistance patterns between geographical regions, where most of these differences were ascribed to antibiotic use regulation. For instance, Australia and New Zealand strictly regulate the use of fluoroquinolones, unlike Asian countries. In another case, *C. difficile* isolates in Australia and New Zealand exhibited high levels of resistance to clindamycin, moxifloxacin, erythromycin, tetracycline, and rifamixin (Lim et al. 2018; Putsathit et al. 2021). Even though tetracycline resistance is not frequently reported in European studies, high levels of resistance to tetracycline and fluoroquinolone in Asia have been associated with the gross overuse of these antimicrobials in animal agriculture (Dingle et al. 2019). This corresponds with the predominant *C. difficile* Clade 5/RT078 (ST11) in Asia, which is known for its resistance to tetracycline (Mengoli et al. 2022). Just as it is suggested that the hypervirulent RT027 evolved due to fluoroquinolone overuse (Singh and Mudey 2021), the spread of RT078 (ST11) may be attributed to the widespread use of tetracycline in animal production (Imwattana et al. 2021).

In the South African agricultural sector, many different classes of antibiotics are available for purchase without the need for a prescription from a veterinarian. For example, tetracycline and sulfonamide antibiotics are under the Stock Remedies Act 36 of 1947, which allows for the distribution without a veterinary prescription. Since the South African livestock industry has unique challenges, it necessitates public access to these antibiotics. For instance, farming practices can be extremely remote, and rural farmers will keep antibiotics on‐site as they might not have access to a veterinarian to consult about the treatment of a diseased animal (Manyi‐Loh et al. 2018; Mokhutsoane and Essack 2023). Veterinarians may prescribe antibiotics to livestock producers to keep on‐site in these remote areas (Farhan et al. 2024), which creates a possibility for antibiotics to be administered at the user's discretion, and hinders effective antibiotic surveillance and stewardship in the South African livestock sector (Mshana et al. 2021). There is limited literature on the distribution and use of antibiotics in food‐producing animals in South Africa, and with no previous research conducted on the prevalence of *C. difficile* in South African livestock, it is imperative to consider investigating potential reservoirs of ABR *C. difficile* in these sectors.

Erythromycin‐resistant *C. difficile* has also been associated with CDI outbreaks (O'Connor et al. 2009). Such resistance in *C. difficile* is attributed to erythromycin ribosomal methylase (*erm*) genes (Tang‐Feldman et al. 2005), which aid in the expression of the macrolide–lincosamide–streptogramin (MLS) B group of protein synthesis inhibitors (Farrow et al. 2000). A study by Imwattana et al. (2021) found that the majority of *C. difficile* isolates harbor *erm*B and *tet*M genes, which aid in its resistance to erythromycin and tetracycline. Furthermore, a recent study conducted in China determined the ABR profiles of 67 *C. difficile* strains by performing an agar dilution on Brucella blood agar, as well as determining ABR genes through PCR. The study indicated that ~57% of nontoxigenic isolates from healthcare environments present with erythromycin resistance (Dang et al. 2024). Erythromycin resistance is also prevalent in animal and environmental isolates, mediated through the presence of MLS‐B resistance genes (Tercero‐Guerrero et al. 2024). Nontoxigenic strains of animal origin frequently carry MLS‐B resistance elements and may act as a reservoir for these resistance genes (Wickramage et al. 2021).

Most *C. difficile* isolates from food‐producing animals are susceptible to vancomycin and metronidazole (Hampikyan et al. 2018; Kecerova et al. 2019; Masarikova et al. 2020; Diniz et al. 2021). However, some studies have indicated a low prevalence of resistance to vancomycin and metronidazole in a small number of agricultural (Blasi et al. 2021) and clinical (Dilnessa et al. 2022) sectors. Therefore, the potential for epidemiologically important *C. difficile* strains to acquire resistance to clinically relevant antibiotics used in CDI treatment cannot be disregarded.

### 2.2 *C. difficile* Environmental and Animal Origin

*C. difficile* can be found throughout the environment as well as in a wide range of animal species, including pigs, cattle, horses, domestic canines and felines, rabbits, polar bears, and nonhuman primates (Kachrimanidou et al. 2019; Weese et al. 2019; Weese 2020). The majority of these animals are asymptomatic carriers and can easily shed *C. difficile* spores into the environment (Redding et al. 2021). Recent studies have also highlighted the presence of toxigenic *C. difficile* strains present in wastewater treatment plants, biosolids, soil, and natural environments polluted by agricultural run‐off or feces (Lim, Androga, et al. 2018; Marcos et al. 2022; Chisholm et al. 2023; Bolton and Marcos 2023; Rui et al. 2024).

Chisholm et al. (2023) found toxigenic *C. difficile* present in 12 wastewater plants in Western Australia. Agricultural slurries often undergo a similar treatment process to that of urban wastewater (Rusănescu et al. 2022) to remove solids, organic matter, and potential pathogens. The resulting effluent or biosolids are often utilized as irrigation or organic fertilizers on crops and animal grazing camps (Marchuk et al. 2023). Where *C. difficile* survives wastewater treatment, it can be present in 0%–82% of treated wastewater effluent, and 73%–100% of treated biosolids (Chisholm et al. 2023). Therefore, it can be expected to observe a high number of pathogens released into the environment where wastewater and biosolids are used as fertilizers in agricultural practices (O'Brien and Hatfield 2019; Illarze et al. 2023).

Lim et al. (2018) isolated *C. difficile* from compost and household lawns in Australia. Isolates indicated resistance to various antibiotics, including those of clindamycin and tetracycline. In a more recent study, Cautivo‐Reyes et al. (2023) investigated *C. difficile* dissemination in the soils of Western Australia and observed that the prevalence of *C. difficile* was higher in densely populated areas. The most prevalent RTs in these areas were RT014/020 and RT010, where the toxigenic RT014/020 is considered the main disease‐causing RT in pigs and humans throughout Australia (Knight et al. 2015; Hong et al. 2020). These findings are similar to previous studies that have detected RT014/020 in soil, compost, and manure (Simango and Mwakurudza 2008; Alam et al. 2014; Janezic et al. 2018; Shivaperumal et al. 2020).

In some cases, household contamination with *C. difficile* spores brought in on the soles of shoes, or through pets, has been shown to be higher (32%) (Shivaperumal et al. 2020) than the transfer within hospitals in the United States (17.1%) (Kanwar et al. 2019). Furthermore, veterinary professionals may still carry the highest number of *C. difficile* spores on the soles of their shoes (86.7%–100%) (Wojtacka et al. 2022).

It has been observed that frequent contact with animals can increase the risk of transmission of pathogens to humans (Furtado et al. 2024). For example, smallholder farms produce on a much smaller scale than commercial enterprises (Davis et al. 2017; Fan and Rue 2020), but are thought to be more likely to contribute to an increased risk of zoonotic transmission (Bandelj et al. 2016). Farmers and farm personnel on smallholder farms are in more frequent, direct contact with their animals than within commercial livestock production systems (Rodriguez et al. 2017).

Conducting a field experiment, Frentrup et al. (2021) observed long‐term contamination through the application of chicken manure to agricultural fields. Diverse *C. difficile* was observed in soil samples only after the fields were fertilized with chicken manure, and the samples remained positive throughout the study. Clinically relevant RT001, RT014, and RT014/020 were observed in manure, fertilized soil, and dust samples. These RTs are some of the most prevalent disease‐causing strains in Europe (Davies et al. 2016). Therefore, the use of biosolids and animal manure as fertilizers contaminates agricultural soil and contributes significantly to the persistence of *C. difficile* in these environments.

#### 2.2.1 Food‐Producing Animals

*C. difficile* is part of the normal microbiota of most mammals; however, it has been known to cause diarrhea in food‐producing animals, such as cattle and pigs (Moono et al. 2016; Blasi et al. 2021). Ranging from subsistence farmers who raise livestock for personal use to mega‐farming operations responsible for the export of meat, South Africa is uniquely structured in terms of its farming practices. Even so, studies investigating the impact of the farm environment on the spread of *C. difficile* have not yet been conducted in South Africa (Marcos et al. 2021, 2023; Blau et al. 2023).

Food‐producing animals in South Africa can be divided into two main categories: ruminants and monogastric animals. Ruminant animals include cattle, sheep, and goats, whereas pigs and poultry make up the majority of the monogastric sector. Due to differences in gastrointestinal physiology, animals can utilize different nutritional sources (Cheng et al. 2022). It can therefore be theorized that a difference in the prevalence, as well as strains of *C. difficile*, may be observed between ruminant and monogastric food‐producing animals.

Knetsch et al. (2018) observed extensive coclustering of certain human and animal *C. difficile* strains, suggesting potential zoonotic spread of *C. difficile*. It has also been observed that the hypervirulent RT078 (ST11) strain has a unique ability to move between livestock and human hosts (Knetsch et al. 2018), has no geographical barriers, and transmission routes may not be limited to a specific community (Spigaglia et al. 2023).

##### 2.2.1.1 Cattle and Small Ruminants

South African cattle production systems can be divided into dairy and beef cattle. Dairy cattle are used for milk production, where either a total mixed ration (TMR)‐based or a pasture‐based production system is applied. Cows kept in TMR‐based production systems receive all their nutrient requirements as a complete feed. These animals are mostly kept indoors and are intensively managed. In contrast, extensive pasture‐based systems rely on the upkeep of good‐quality pastures as the main source of nutrition for their animals, and cows may receive supplementary feeding where needed. Pasture‐based systems also require quite intensive management, as it demands a high input to maintain a favorable profit (Galloway et al. 2024). Beef cattle can also be categorized into similar production systems. Feedlots are intensive TMR‐based feeding facilities where cattle are fed for meat production, whereas extensive beef production systems rely on natural vegetation as the animal's main source of nutrition (Spies 2011).

The prevalence of *C. difficile* in small ruminants, such as sheep and goats, has not been widely reported. Previous studies have mainly focused on carcass contamination of sheep and goats; however, the limited studies available on *C. difficile* in small ruminant feces vary widely. Avberšek et al. (2014) observed *C. difficile* in almost 6% of sheep and up to 9.8% in goats, and observed clinically relevant RT010 and RT014/020 to be associated with small ruminants in Slovenia. Furthermore, an RT078 *C. difficile* isolate was obtained from a Nigerian dwarf goat in a Dutch zoo (Álvarez‐Pérez et al. 2014). *C. difficile* infections are not a relevant disease in small ruminants; however, the presence of hypervirulent *C. difficile* strains may contribute to the potential of zoonosis or contamination in the food chain.

Even though the cattle reservoir of *C. difficile* is still greatly debated, a high prevalence (14%–60%) of *C. difficile* on dairy farms appears to be consistent throughout the literature (Masarikova et al. 2020; Redding et al. 2021; Spigaglia et al. 2023; Cuperus et al. 2024). Pasture‐based systems also often make use of manure or dairy effluent as fertilizer on pastures (O'Brien and Hatfield 2019; Illarze et al. 2023), and may contribute to the pathogen burden on these farms (Illarze et al. 2024). Since *C. difficile* is known to survive wastewater treatment (Chisholm et al. 2023), it can be theorized that the application of dairy effluent may contribute to environmental contamination and dissemination of *C. difficile* on agricultural lands (Lim, Androga, et al. 2018; Rui et al. 2024). In 2007, local water supply in Finland was contaminated with treated sewage effluent, which resulted in the first recorded waterborne transmission of *C. difficile* (Kotila et al. 2013).

Calves younger than 4 weeks of age have the highest prevalence of *C. difficile* within a dairy herd, which corresponds with studies where calves were identified as carriers (Masarikova et al. 2020; Redding et al. 2021; Blasi et al. 2021; Shurson et al. 2022). The bacterium has been detected in both diarrheic and healthy calves (Hammitt et al. 2008; Jessop et al. 2024), of which the prevalence thereof can also range widely (2%–60%) (Cho and Yoon 2014). In contrast, Spigaglia et al. (2023) observed that the prevalence of *C. difficile* may remain constant within a herd, regardless of the age of the animal. Inconsistent findings across the literature regarding the prevalence and the associated animal age group most likely associated with *C*. difficile remain.

The most frequent *C. difficile* RTs associated with calves include RT033, RT078, and RT126 (Masarikova et al. 2020; Spigaglia et al. 2023). Cuperus et al. (2024) observed that almost 20% of dairy farms harbor potential zoonotic *C. difficile* RTs. Hammitt et al. (2008) noted that the human–pathogenic RT078 tends to be predominant in diarrheic calves. This is further supported by Rodriguez‐Palacios et al. (2006), who observed the hypervirulent RT027 and clinically important RT017 in Canadian dairy calves, and Blasi et al. (2021), who observed RT078 and RT126 in Italian calves. Even though Weese (2020) emphasized that no *C. difficile* outbreaks have been reported in calves, toxigenic strains and human–pathogenic RTs are still frequently detected in these animals (Rodriguez‐Palacios et al. 2006).

A study by Redding et al. (2021) suggested that the zoonotic transfer of *C. difficile* to personnel on dairy farms is rare. Similar findings contribute to the debate of cattle as a reservoir for *C. difficile* (Rodriguez et al. 2017); however, the risk to public health remains. The human–pathogenic RTs observed in dairy production systems are of One‐Health concern, as some of these RTs have been on the rise in the environment and CA‐CDI cases (Knight et al. 2016; Androga et al. 2018; Janezic et al. 2018; Azimirad et al. 2020; Tsai et al. 2022).

A much lower prevalence of *C. difficile* is associated with beef cattle (3%–18%) (Weese 2020). *C. difficile* was only detected in 3% of all incoming animals entering feedlots, and a slight increase in the detection of *C. difficile* was observed as the animals aged (Costa et al. 2012). The spread of disease in feedlots is a major concern for profitability, production, and animal welfare (Cameron and McAllister 2016). Various cattle‐associated pathogens have been known to spread through dust, feces, and contact surfaces (Koyun et al. 2023). Since *C. difficile* has a unique ability to persist in the environment through spores, it is plausible that the slight increase in prevalence could be attributed to its potential dispersal throughout a feedlot system.

##### 2.2.1.2 Pigs

The overall prevalence of *C. difficile* in pigs can range from 8% to 61% (Keessen et al. 2011a; Kim et al. 2018). Mature pigs are mostly asymptomatic carriers of this bacterium (Knetsch et al. 2014); however, a higher prevalence and shedding rate are still associated with piglets and diarrheic pigs (Weese 2020). The hypervirulent *C. difficile* RT078 is associated with pigs worldwide (Goorhuis et al. 2008; Squire et al. 2013; Kim et al. 2018; Knetsch et al. 2018; Moloney et al. 2021); however, the emerging RT033 is becoming more prevalent in both swine production and clinical cases. *C. difficile* RT033 indicates an ability to adapt to food‐producing animal hosts, as well as animal production system environments (Spigaglia et al. 2023; Bandelj et al. 2016). This could be attributed to some pig‐associated RT033 strains that harbor genomic traits similar to RT078, enhancing their environmental fitness and pathogenic potential (Alves, Cano, et al. 2022).

In 2008, RT078 was suggested as an emerging *C. difficile* strain in both humans and pigs (Goorhuis et al. 2008). However, a 14‐year surveillance study in the Netherlands found no evidence of coclustering of human and pig RT078 isolates (van Dorp et al. 2017). This has not been the case in Ireland and some European countries, where extensive genomic analysis of human and pig RT078 revealed probable transmission and a close overlap between human and pig isolates (Moloney et al. 2021). Furthermore, Keessen et al. (2013) found that the majority of people who have daily, direct contact with pigs were colonized with identical isolates to those detected in the corresponding pig production facilities.

Even though *C. difficile* is mainly thought to be transmitted through the fecal–oral route, other potential transmission routes should not be disregarded. Airborne dispersal of *C. difficile* spores has been observed in pig production systems (Keessen et al. 2011b), and multiple studies pertaining to livestock (Lim, Androga, et al. 2018; Knight and Riley 2019). Airborne transmission is not uncommon, even in hospital settings, with spores detected in some clinic air vents (Best et al. 2010). On average, 66% of air filters in pig houses contain *C. difficile* spores (Alves, Cano, et al. 2022). Dust, water, contact surfaces, and other parts within a pig production facility may harbor *C. difficile* spores and so contribute to the transmission thereof. Still, conflicting literature on pigs as potential reservoirs and risks to public health persists. Therefore, the true burden of *C. difficile* detected in pig production systems is yet to be determined.

##### 2.2.1.3 Poultry

Very limited research is available on the prevalence of *C. difficile* in poultry, as most studies focus on poultry meat and associated products. Even so, the bacterium has been detected in poultry manure intended for use as organic fertilizer (Frentrup et al. 2021). *C. difficile* is detected at a low prevalence (2.3%) in broiler chickens (Harvey et al. 2011a); however, in Zimbabwe, *C. difficile* could be detected in 29% of broiler chicken feces (Simango and Mwakurudza 2008). A more recent study indicated an intermediate prevalence (17%) of *C. difficile* in feces of native poultry in Iran (Barezi et al. 2023). Almost 90% of the duck, partridge, quail, and native chickens harbored toxigenic RT027 and RT078 *C. difficile* strains, and all the isolates were resistant to ampicillin. Here, a concerning 28% of *C. difficile* isolates indicated resistance to metronidazole; however, none of the isolates exhibited resistance to vancomycin. Hypervirulent RT027 and RT078 isolates of animal origin with resistance to clinically relevant antibiotics highlight the unique epidemiological abilities of *C. difficile*.

Albeit limited literature is available on *C. difficile* from poultry animals and ‐environments, findings suggest that these animals may contribute to the pool of ABR *C. difficile*. These resistant *C. difficile* isolates not only pose a threat to food safety but also considerably contribute to the threat of *C. difficile* as an emerging pathogen within a One‐Health approach.

#### 2.2.2 Wildlife and Game

*C. difficile* has been isolated from a wide range of wildlife, including polar bears in the Canadian Arctic (Weese et al. 2019). However, very few studies have been able to isolate *C. difficile* from deer (Arryo et al. 2005; French et al. 2010). A singular study on *C. difficile* in animals in the southern parts of Africa was conducted in Zimbabwe (Simango and Mwakurudza 2008), and did not consider game species intended for human consumption.

The unique fauna and flora of South Africa enable cograzing of food‐producing animals and wildlife, which can result in fecal–oral pathogen transfer between the animal species (van den Honert et al. 2021). A similar observation was made in an earlier review, where *C. difficile* strains in wildlife often reflect the degree of cohabitation or exposure to human settlements (Weese 2020). This is easily plausible, as densely populated areas have been shown to harbor high levels of *C. difficile* in the environment (Cautivo‐Reyes et al. 2023; Marchuk et al. 2023).

Similar to urban wildlife exhibiting human‐associated *C. difficile* RTs, livestock‐associated RT078 is primarily detected in wildlife with close proximity to farm environments (Andrés‐Lasheras et al. 2017). Observations by de Oliveira et al. (2018) confirm that regular farm wildlife, such as rodents, may carry livestock‐associated *C. difficile* RTs. The idea of rodents being vectors for the dissemination of *C. difficile* is further supported by previous studies (Jardine et al. 2013; Firth et al. 2014; Rothenburger et al. 2017), and again highlighted in a recent study by Azimi et al. (2024), where most of the *C. difficile* isolates were obtained from rats trapped within the city center of Tehran.

Even though humans may come into close contact with zoo animals, the role these animals play as potential reservoirs for *C. difficile* remains understudied. The toxigenic *C. difficile* RT014/020 has been isolated from zoo animals, including Asian elephants, cheetahs, pheasants, and kudu (Alam et al. 2019). Similarly, about 3% of zoo animals were found to be colonized with the human–pathogenic RT078, and a metronidazole‐resistant isolate was obtained from a Zebra foal in a study conducted by Álvarez‐Pérez et al. (2014).

Ostriches are mostly farmed commercially for the export of feathers and meat; however, they are still considered game (D'Amato et al. 2013). *C. difficile* prevalence has been reported as 20%–60% in these birds, where up to 70% may be toxigenic strains (Jafari et al. 2017). It has been theorized that *C. difficile* may contribute to gastrointestinal disturbances in wildlife (Zhen et al. 2022). Even though a higher prevalence is associated with younger chicks (Shivaprasad 2003; Jafari et al. 2017), only one outbreak of CDI in 9‐day‐old ostrich chicks has been documented (Frazier et al. 1993). Nonetheless, the prevalence of *C. difficile* in ostriches is still much higher compared with other poultry animals.

#### 2.2.3 Companion Animals

##### 2.2.3.1 Horses

Some of the earliest identifications of *C. difficile* in domestic animals involved horses, where the presence of the bacterium was commonly associated with diarrheic foals (Ehrich et al. 1984; Jones et al. 1987). Even though the prevalence of *C. difficile* differs widely in the literature (2.4%–40%) (Båverud et al. 2003; Kecerova et al. 2019; Hain‐Saunders et al. 2022), various studies have shown that diarrhea and acute colitis are potential indicators of a CDI (Diniz et al. 2021; Weese et al. 2021; Hain‐Saunders et al. 2022). Several *C. difficile* RTs (i.e., RT001, RT012, RT014, and RT017) are associated with horses (Rodriguez et al. 2014b; Uchida‐Fujii et al. 2023), and coinfections of *C*. *difficile* and *Clostridium perfringens* are common in neonatal foals, often resulting in mortalities (Magdesian et al. 2002, 2022; Uzal et al. 2012). Other toxigenic *C. difficile* strains of importance, such as the hypervirulent RT027 and livestock‐associated RT078, can sometimes be observed in both healthy foals and mature horses (Weese 2020).

In humans, previous antibiotic exposure is considered a high‐risk factor for developing a CDI (Slimings and Riley 2014). However, the literature on previous antibiotic exposure as a similarly high‐risk factor in horses remains inconsistent. The majority of studies are able to determine that CDIs occur more frequently in horses with a history of antibiotic use (Båverud et al. 2003; Morsi et al. 2019; Hain‐Saunders et al. 2022; Uchida‐Fujii et al. 2023); however, there is no consistent set of parameters that may induce a CDI outbreak among horses. Similar to humans, it is theorized that the intestinal microbiota of individual animals may be a crucial component in determining susceptibility to a CDI (Kachrimanidou et al. 2019).

##### 2.2.3.2 Household Pets

Companion animals are unique in terms of their closeness to humans, many of which coinhabit the same households. Williamson et al. (2023) observed transmission of *C. difficile* between humans, canines, and the environment. Transmission follows a fecal–oral route and is easily attainable due to the close coinhabitation of man and canine. The coclustering of *C. difficile* strains provides evidence that support the theory of zoonotic transmission between animals and humans (Redding et al. 2022), however, the role of companion animals are often overlooked. Bjöersdorff et al. (2021) were able to determine that virulent strains of *C. difficile* can be exchanged between dogs and humans. These strains also indicated resistance to clindamycin, erythromycin, and tetracycline, which are antibiotics frequently used in veterinary medicine. The resistance of *C. difficile* to these antibiotics is consistent across the literature, where clindamycin and erythromycin resistance is frequently reported, while tetracycline resistance is less frequent in canines and felines (Lim, Androga, et al. 2018; Teng et al. 2019; Dingle et al. 2019; Putsathit et al. 2021; Mihajlov et al. 2024). Clindamycin is still commonly used to treat bacterial infections in dogs and cats (Mercer 2022), whereas tetracycline is more commonly used to treat livestock‐associated infections.

#### 2.2.4 Food Processing Environments

Almost 50% of abattoir samples have been found to harbor *C. difficile* (Marcos et al. 2021). Asymptomatic animals entering the abattoir lairage area may carry various *C. difficile* strains, and the added stress of transport can increase fecal shedding of bacterial pathogens. The majority of *C. difficile* RTs detected in transporting trucks and abattoir lairage areas, such as RT010, RT078, and RT126, are associated with CDIs in humans (Álvarez‐Pérez et al. 2018). Furthermore, the pathogen has also been detected in slaughter lines (20%–70%) (Harvey et al. 2011b; Álvarez‐Pérez et al. 2018) and abattoir wastewater (20%) (Abay et al. 2022).

Even though a low prevalence of *C. difficile* has been detected in abattoirs in Iran, a moderate prevalence (3%–28%) has been reported in meat processing (Esfandiari et al. 2014). This is consistent with previous literature, where a low prevalence (9%) of *C. difficile* was detected in sausage manufacturing plants in the United States (Harvey et al. 2011b). Both animal fecal matter and water can contribute to the contamination of the abattoir environment. Therefore, special care should be taken when animals are eviscerated, as beef, sheep, and poultry have shown increased carcass contamination post‐evisceration (Marcos et al. 2021).

### 2.3 Emerging Foodborne Pathogen

*C. difficile* has been detected in raw meat, fish, fermented sausages, leafy greens, coleslaw, restaurant side dishes, as well as in soft cheeses and other dairy products (Lim et al. 2020; Borji et al. 2023; Hazarika et al. 2023). A high prevalence is also consistent across the literature with fish and seafood products (Metcalf et al. 2011; Romano et al. 2018; Agnoletti et al. 2019), plants (Romano et al. 2018; Marcos et al. 2021), RTE foods (Tkalec et al. 2020), and dried products (Rodriguez et al. 2021). Even so, the detection thereof is sporadic, and no epidemiological patterns linked to food products can be derived from current literature.

#### 2.3.1 Animal‐Based Products

*C. difficile* has been identified in various food products, where it has mostly been associated with meat and seafood. Animal carcasses are thought to be contaminated through fecal exposure and improper slaughtering practices (Licciardi et al. 2021; Abay et al. 2022) since *C. difficile* can also be detected in animals prior to slaughter (Keessen et al. 2011a). However, *C. difficile* is not only limited to meat products and can be found in a wide variety of animal‐based products, including dairy‐based products and animal hides (Bolton and Marcos 2023; Borji et al. 2023; Rui et al. 2024).

A recent study in Canada observed that all *C. difficile* isolated from retail meat were comparable to isolates from clinical cases (i.e., RT027, RT106, and RT131), indicating that retail meats could potentially harbor *C. difficile* and have the potential to contribute to CA‐CDI (Tan et al. 2022). This theory is supported by various other studies from Brazil (Pires et al. 2018), Italy (Licciardi et al. 2021), India (Hazarika et al. 2023), Ireland (Bolton and Marcos 2023), Iran (Bacheno et al. 2022), and some Asian countries (Esfandiari et al. 2021). Furthermore, Rodriguez et al. (2014a) determined RT078 and RT014 to be predominant in beef and pork retail meat in Belgium. However, no CDI outbreaks could directly be traced back to these food products.

Poultry meat products have an overall *C. difficile* prevalence of 6.2% (Borji et al. 2023), and the detection thereof can range between 3% and 70% (Taha Attia 2021; De Boer et al. 2011; Guran and Ilhak 2015; Rodriguez et al. 2017; Rodriguez et al. 2016). Literature on the specific *C. difficile* RTs associated with poultry meat products is scarce, but some studies have detected RT001 (Abdel‐Glil et al. 2018; Barezi et al. 2023; Rui et al. 2024). Furthermore, *C. difficile* isolates from a study by Taha Attia (2021) observed resistance to the clinically important antibiotic metronidazole, as well as several other antibiotics, including tetracycline, clindamycin, and moxifloxacin.

*C. difficile* RT033 has further become predominant in swine infections (Alves, Nunes, et al. 2022) and is also on the rise in CDI cases in humans (Androga et al. 2018). *C. difficile* RT126, which is genetically close to the livestock‐associated hypervirulent RT078 (Álvarez‐Pérez et al. 2017), has now become the main RT associated with certain healthcare settings in Iran (Azimirad et al. 2020). While also recently being detected in river water (Tsai et al. 2022), the changing epidemiology of *C. difficile* RT126 threatens to become a large contributor to CA‐CDIs (Liu et al. 2023).

Although the common livestock‐associated RT078 was not detected in the study of Knight et al. (2016) where the contamination of veal carcasses were investigated, a sublineage RT127 was observed as one of the major strains contributing to the carcass contamination. This RT is becoming more frequent in hospital settings and has also been detected in seafood in Taiwan (Huang et al. 2021).

The hypervirulent RT027 is frequently found in processed meat products, including salami, processed meat sausages, and uncooked meatballs (Muratoglu et al. 2020). Even so, *C. difficile* contamination within meat processing plants varies widely. Curry et al. (2012) detected RT078 in only 2% of minced meat, even though 52% of samples taken within the processing facility delivered positive *C. difficile* results. Similarly, even though *C. difficile* was found on the hides of beef cattle entering abattoirs; no contamination of carcasses or the end ground‐beef product was observed (Kalchayanand et al. 2013).

Toxigenic *C. difficile* (RT078 and RT011/018) has been isolated from raw bovine milk in Italy (Romano et al. 2018) and buffalo milk in Iran (Rahimi et al. 2014). It has also been detected in soft cheeses (Marcos et al. 2021); however, *C. difficile* is not often common in dairy products. Interestingly, *C. difficile* has not yet been isolated from honey or other bee products (Wojtacka et al. 2020). It is theorized that since honey exhibits some bactericidal activity against *C. difficile*, it can inhibit spore proliferation (Yu et al. 2020). This may indicate that natural alternatives to conventional controls of *C. difficile* could potentially be of use in food processing environments.

For many South Africans, game meat (venison) forms a large part of their diet and culture. Game farming is one of the fastest growing sectors in the agricultural industry, where *Bovidae* (antelope) and *Cervidae* (deer) species, as well as *Equus quagga burchellii* (zebra) are hunted either for export, or on privately owned game reserves for consumption by the individual (Gouws et al. 2017b). Ostrich, even though mostly farmed commercially, is in some cases also considered venison (Kudrnáčová et al. 2018). Whether the term “venison” can be used to describe game meat still remains largely debated in the literature. Kudrnáčová et al. (2018) urge to distinguish between game meat and venison, where game meat is considered meat from wild game animals and venison from farmed game animals (i.e., deer or springbuck farmed for harvesting) (D'Amato et al. 2013). However, some define venison as meat exclusively from *Cervidae*, and game meat referring to any other game species, while others consider all game meat to be venison. In this review, the term “venison” will refer to meat from game either harvested for commercial purposes or hunted for personal use. Ostrich and game bird meat is excluded from this category and will be referred to as “Ostrich” and “game meat” or uniquely defined where necessary.

The South African Department of Forestry, Fisheries and the Environment (DFFE) has recently proposed a Game meat strategy, with the hope of further establishing a strong local market (DFFE 2022). Strategic objectives of the strategy include transforming the industry through skills, knowledge, and education, as well as enabling the development of large game production and the associated value chain in community‐owned areas. This will hopefully contribute to the local venison market and push for stricter South African regulations regarding hunting, processing, and sale of venison products.

Food safety in relation to venison can be categorized into two systems in South Africa. First, a strictly regulated hunting/harvesting system with the intent for product export, and second, an unregulated system where venison is intended for personal use (van der Merwe et al. 2014). Since game animals are hunted and eviscerated in the veld, certain factors may contribute to the degree of carcass contamination. From shot placement to carcass dressing, the knowledge of correct hunting and slaughtering practices can play a pivotal role in ensuring the safety of the meat (van der Merwe et al. 2014; Giuggioli et al. 2017). Even though *C. difficile* is not routinely tested for in food products, *C. perfringens* is included in routine hygiene testing in Europe, as it is an associated foodborne pathogen of animal origin (Bendary et al. 2022). Coinfection of *C. perfringens* and *C. difficile* has been observed in some animals (Uzal et al. 2012) and humans (Forero et al. 2019), as these organisms are both strict anaerobes that can naturally colonize the gastrointestinal tract (Li et al. 2023). Therefore, if *C. perfringens* is present within a fecal or food sample, it is possible that *C. difficile* will also be present.

Currently, there are no known studies that have investigated the prevalence of *C. difficile* in South African venison; most studies, however, focus on the detection of *C. perfringens*. European game meat hygiene studies detected *C. perfringens* in wild boar, red deer, and roe deer (Gomes‐Neves et al. 2021). *Clostridium* spp. has frequently been detected in venison, such as springbuck (*Antidorcas marsupialis*) and black wildebeest (*Connochaetes gnou*) (Gouws et al. 2017a; Gouws et al. 2017b). Furthermore, *Clostridium* species has also been detected, and associated with mortality in ostriches (Shivaprasad 2003; Videvall et al. 2020). Therefore, the detection of such *Clostridium* spp. may hint towards the presence of *C. difficile* in these meat products.

*C. difficile* has been associated with pigs, a trend that is reflected in the European wild boar population as well (Giuggioli et al. 2017; Darwich et al. 2021). However, even though *C. difficile* has been isolated from captive deer and antelope (Arryo et al. 2005; Álvarez‐Pérez et al. 2014; Alam et al. 2014), but it has not yet been studied in venison intended for human consumption.

#### 2.3.2 Plants and Plant‐Based Products

There is some information available on *C. difficile* in animal‐based products, with very little available on leafy greens and plant‐based products. However, the hypervirulent RT027 and human–pathogenic RT078 has been detected in various plant products, including RTE salads (Rahimi et al. 2015), and vegetables (Candel‐Pérez et al. 2019; Bolton and Marcos 2023). Even so, the detection of *C. difficile* in salads and fresh vegetables remains low throughout the literature (1%–5.5%) (Metcalf et al. 2010; Usui et al. 2020; Scholtzek et al. 2022; Borji et al. 2023). *C. difficile* has been detected at much higher percentages in root vegetables in Germany (26%), Australia (10%–30%), and Slovenia (18%) (Lim, Foster, et al. 2018; Tkalec et al. 2019; Scholtzek et al. 2022).

Interestingly, plant‐derived phenolic compounds have been observed to either aid the activity of antibiotics or to be effective bactericidal agents in vitro. Curcumin, a compound of turmeric, is thought to inhibit *C. difficile* growth, but indicates no sporicidal activity (Mody et al. 2020). The bacteriostatic nature of curcumin is further supported by Wultańska et al. (2024) and Roshan et al. (2017), who found *C. difficile* strains to also be susceptible to trans‐cinnamaldehyde. Peppermint oil, allicin (garlic), menthol, zingerone, and honey have also indicated promising antibacterial activities against *C. difficile* (Roshan et al. 2017) and are able to reduce overall toxin production (Roshan et al. 2018).

RTE foods are known for various foodborne pathogen contamination; however, *C. difficile* contamination in RTE salads has been consistently low (1%–7.5%) across the literature (M. M. Bakri et al. 2009; Yamoudy et al. 2015; Romano et al. 2018). RTE salads often harbor clinically relevant RT001 and RT017, classifying them as a high‐risk *C. difficile* food group. *C. difficile* RT001 and RT010 have also been detected in vegetable samples in Slovenia (Tkalec et al. 2020), and RT010, RT014, RT023, and RT078 are frequently associated with salads and vegetables (Scholtzek et al. 2022). Further research into the exact contributor and point of contamination of RTE salads and vegetables should be considered.

#### 2.3.3 Potential for Foodborne Transmission

Lim et al. (2020) described the role an effective One‐Health approach may play towards the control of CA‐CDI, emphasizing the possible transmission routes the bacterium may follow in the environment. Although most CDI outbreaks are associated with hospitals (Muto et al. 2007; Wong‐McClure et al. 2012; Oleastro et al. 2014), some outbreaks have been linked to water (Kotila et al. 2013). It has also been proven that some food additives can enhance the virulence of the bacterium (Collins et al. 2018). Historically, environmental sources of *C. difficile* were only identified due to similar bacterial strains and antibiotic resistance profiles, present in both farm environments, food‐producing animals, food products, and clinical isolates (Bolton and Marcos 2023). However, to date, no outbreaks have directly been linked to food products or food‐producing animals (Lim et al. 2020).

Potential foodborne transmission of *C. difficile* is supported by many studies, as high detection rates have been observed in many different food groups (Kong 2020). Since *C. difficile* is classified as obligate anaerobes, it is more plausible that transmission relies on spore contamination rather than vegetative cells. *C. difficile* spores are able to persist in extreme conditions and cannot be destroyed by freezing, desiccation, high cooking temperatures, and common disinfecting agents. It is therefore safe to theorize that spores can spread through contaminated environments and food products despite cooking, chilling, and cleaning.

*C. difficile* can persist in modified‐atmospheric packaging (Fatma and Gücükoğlu 2017). Other studies supporting these findings further detected *C. difficile* persisting in high‐barrier vacuum packs. Furthermore, where temperature abuse of these products was observed, a subsequent increase of *C. difficile* by 5log_10_ CFU/g could be seen (McSharry et al. 2021). This suggests that modified‐atmospheric packaging, canning, anaerobic packaging, and vacuum packaging may contribute to the persistence of *C. difficile* in food products. The breakdown of the cold chain may further exasperate the situation (Bouttier et al. 2010; Fatma and Gücükoğlu 2017). Even so, research on the potential foodborne transmission routes of *C. difficile* remains understudied. Since no direct link has been made to food as an outbreak source of CDIs, it is a hindering factor in epidemiological studies. It is therefore necessary to gather information on the nature of this pathogen in various environments (i.e., agriculture, food, packaging, etc.) to fully understand its distribution.

## 3 Conclusion

*C. difficile* is considered a major threat to human health, mostly due to its multidrug‐resistance characteristics. Previously, it was only associated with nosocomial pathogens and antibiotic‐associated diarrhea; the rise in CA‐CDI indicates a potential epidemiological change. Patients with CDIs no longer require having a history of the usual predisposing factors to contract the infection. This may also be attributed to an increased virulence of *C. difficile* strains previously associated with the environment or animals.

The unique ability of *C. difficile* to persist in unfavorable environments due to its spore‐forming nature largely contributes to its transmission as a potential zoonotic and foodborne pathogen. Spores are present in soil, water, animal feces, dust, contact surfaces, and animals. Some *C. difficile* strains are more clinically significant than others, and it is evident that not all strains are limited to a specific animal or food group. Only one study has been conducted in Zimbabwe, where *C. difficile* was observed in chicken meat, chickens, and soil. *C. difficile* strains detected in meat do not always correspond with those found in other food‐producing animals, where meat products may harbor human–pathogenic RTs. Furthermore, hypervirulent *C. difficile* strains present in RTE salads and vegetables support the theory that environmental and specific farming practices contribute to foodborne transmission.

Animal agriculture and the historic use of antibiotics have been thought to contribute to the current resistance profiles observed worldwide. This is evident across regions, as seen in *C. difficile* resistance patterns. Antibiotic resistance is observed in various degrees to lincosamide, fluoroquinolone, rifamycin, tetracycline, beta‐lactam, macrolide, and carbapenem antibiotic classes. Even though resistance to antibiotics used to treat CDIs remains low, the risk of *C. difficile* acquiring resistance to vancomycin, metronidazole, and fidaxomicin persists.

With the rise in CA‐CDIs, alternative transmission routes of *C. difficile* should be considered. The only confirmed CA‐CDI outbreak could be linked to contaminated tap water. There is evidence that farm environments and animals contribute to the spread of CA‐CDI; however, its foodborne transmission is yet to be confirmed. Beyond nosocomial CDI, there is currently no information on the prevalence and dissemination patterns of *C. difficile* in livestock, the environment, wildlife, and game in South Africa. Therefore, establishing routine surveillance of CDI incidence (nosocomial and CA‐CDI cases), together with characterization of *C. difficile* isolates from clinical, environmental, animal, and associated food sources, is necessary to improve prevention and response strategies in South Africa's healthcare system.

## Author Contributions

**M. Gouws:** investigation, writing – original draft, methodology, writing – review and editing, formal analysis, data curation, visualization. **P. E. Strydom:** supervision, writing – review and editing, validation. **D. Rip:** conceptualization, funding acquisition, writing – review and editing, validation, visualization, project administration, supervision, resources, methodology.

## Ethics Statement

The authors have nothing to report.

## Conflicts of Interest

None declared.

## Data Availability

The authors have nothing to report.
